# Visually cued fear conditioning test for memory impairment related to cortical function

**DOI:** 10.1002/npr2.12146

**Published:** 2020-10-21

**Authors:** Kazuya Kuboyama, Yuki Shirakawa, Koji Kawada, Naoki Fujii, Daiki Ojima, Yasushi Kishimoto, Tohru Yamamoto, Maki K. Yamada

**Affiliations:** ^1^ Department of Neuropharmacology Kagawa School of Pharmaceutical Sciences Tokushima Bunri University Sanuki city Kagawa Japan; ^2^ Department of Molecular Neurobiology Faculty of Medicine Kagawa University Kita‐gun Kagawa Japan; ^3^ Department of Biophysics Kagawa School of Pharmaceutical Sciences Tokushima Bunri University Sanuki city Kagawa Japan; ^4^ Present address: Department of Developmental and Regenerative Neurobiology Institute of Brain Science Nagoya City University Graduate School of Medical Sciences Nagoya Japan

**Keywords:** fear conditioning, gratings, magnocellular, neocortex, visual cortex

## Abstract

**Aim:**

Fear conditioning tests are intended to elucidate a subject's ability to associate a conditioned stimulus with an aversive, unconditioned stimulus, such as footshock. Among these tests, a paradigm related to precise cortical functions would be increasingly important in drug screening for disorders such as schizophrenia and dementia. Therefore, we established a new fear conditioning paradigm using a visual cue in mice. In addition, the validity of the test was evaluated using a genetically engineered mouse, heterozygous deficient in *Mdga1* (*Mdga1*+/‐), which is related to schizophrenia.

**Results:**

Mice were given footshocks associated with a visual cue of moving gratings at training in 25‐minute sessions. The mice showed the conditioned response of freezing behavior to the visual stimulus at testing 24 hours after the footshocks. In the test for validation, the *Mdga1*+/‐ deficient mice showed significantly less freezing than wild‐type mice.

**Conclusion:**

The visually cued fear conditioning paradigm with moving gratings has been established, which is experimentally useful to evaluate animal cortical functions. The validity of the test was confirmed for *Mdga1*‐deficient mice with possible deficiency in cortical functions.

## Abstract 2

The visual stimulus of moving gratings could be distinguished from still gratings by mice, and moving gratings were related to footshock. The defect obtained here by low‐frequency grating in MDGA1 deficient mice might reflect that the coarse gratings are less detectable in visual perceptions of the schizophrenic brain.

## Graphical abstract

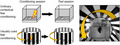

**Abbreviations**
CS: Conditioned stimuli
FC: Fear conditioning
SD: standard deviation
SEM: standard error of the mean
US: Unconditioned stimuli
VFC: Visually cued fear conditioning

## 1 INTRODUCTION

Fear conditioning (FC) describes a learning and memory paradigm in which an animal's ability to acquire and retrieve an association between a conditioned stimulus (CS), such as a place in contextual FC or tone in auditory FC, and an unconditioned stimulus (US), such as footshock or corneal air puff, is assessed. Fear conditioning memory is indexed by quantitation of a behavioral response, such as time spent freezing, which is a behavioral response to fear in rodents and is defined commonly as remaining motionless with the exception of breathing.

Experimentally, FC has advantages of rapid acquisition and high reproducibility and is an objective quantitative behavioral output measure. The short time period required to instill reproducible conditioning, particularly with single‐training trial paradigms, is highly advantageous for studies examining the time course of molecular changes with biochemical, genetic expression (mRNA hybridization), and protein expression (immunolabeling) methods.

Neurologically intact rodents readily exhibit FC with various CSs. However, a distinctly visually cued FC (VFC) system employing freezing as the assessed conditioned response has not been reported. Recently, mainly in the neocortex, researchers have developed approaches to examining animals' brain activity patterns with in vivo imaging during learning. Furthermore, plasticity in the dendritic spines within minutes has been observed in the neocortex.^1^ However, the time courses of the most tasks involving cortical functions have been on the order of many hours or days.

The aim of the present study was to develop a relatively short‐term task in mice associating the US with a specific visual CS, separately to the nonspecific context in pre‐CS with standing gratings. In addition, the test was successfully used to clarify the memory deficit in mice with genetic mutations in a molecule, MAM domain‐containing GPI anchor protein 1 (MDGA1), suggested to be a schizophrenia susceptibility gene.^2^

## 2 MATERIALS AND METHODS

### 2.1 Animals

All experimental procedures were carried out in accordance with the Institutional Guidelines and were approved by the Animal Experiment Committee of Tokushima Bunri University. Mice were maintained on free access to both food and water under a 12‐hour light‐dark cycle. Young adult C57BL/6N mice (8 weeks old; Charles River) were used for conditioning experiments. Before the conditioning, each mouse was subjected to four to six 1‐min handling sessions over 2‐4 days until no signs of fear (eg, excrement) were apparent during handling. Female mice with a heterogenic deficiency in the MDGA1 molecule (C57BL/6N background^3^) were screened for the mutation by PCR using three new sets of primers, which enabled a one‐tube reaction for genotyping (Fwd: GTTCAGCACCCTCGGTGGAC; Mut Rev: CCAGGGTTTTCCCAGTCACG [product size 445 bp for the mutant allele]; Wild Rev: GAGACCTTACCCCGGCATCC [product Size 609 bp for the wild allele]).

### 2.2 VFC

**FIGURE 1:**
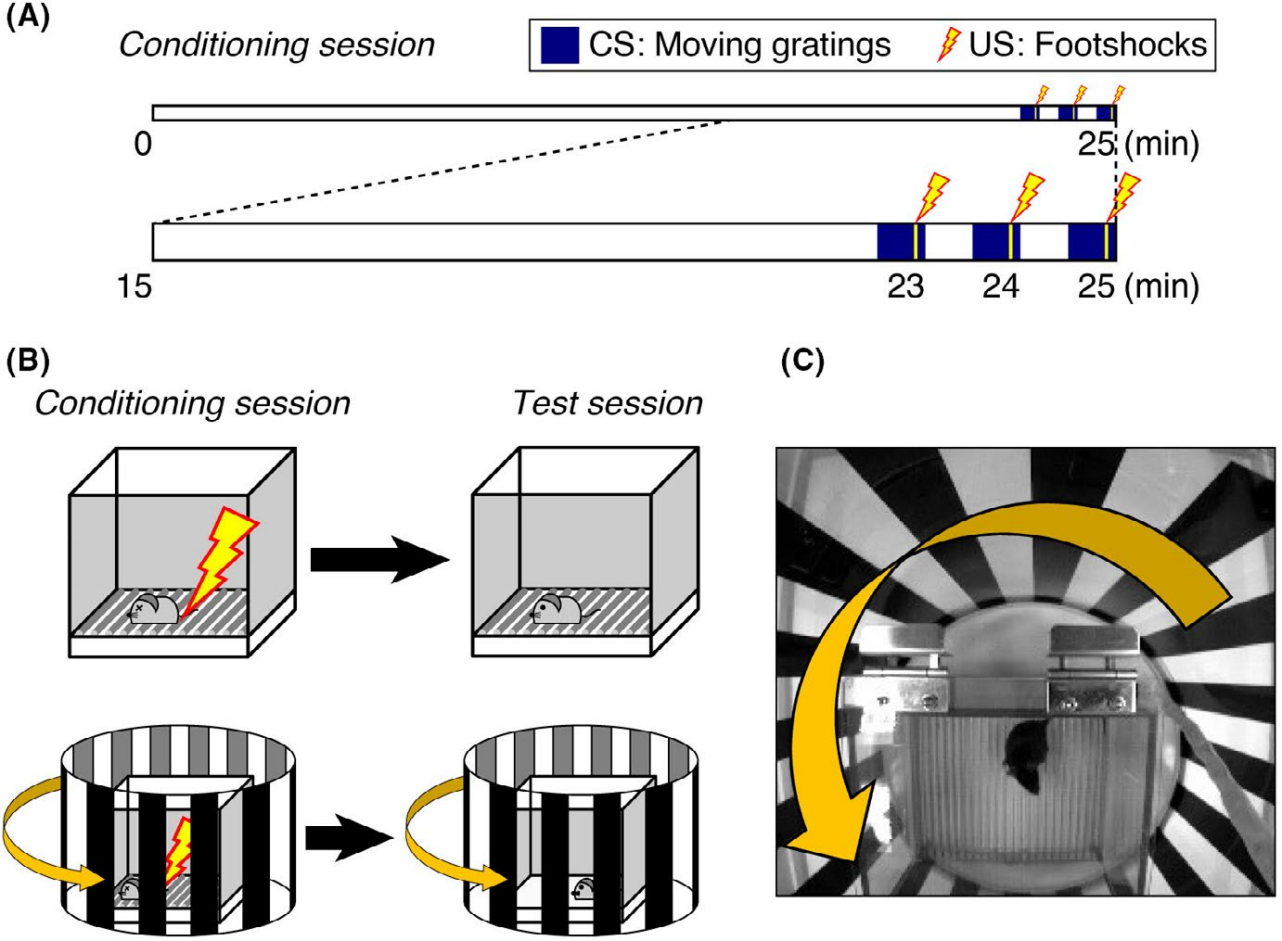
Conditions of visually cued FC of mice. A, Schematic time course of the 25‐min conditioning session. Mice were kept in the chamber 22.5 min prior to CS exposure (grating movement) to allow them to become familiar with the standing gratings and general environment of the box. For CS training, the gratings were moved from right to left (0°) for 30 s, and 2‐s footshock was delivered at 23‐25 s within the 30‐s period. This pairing was repeated three times with 30‐s inter‐pairing intervals. B, Ordinary contextual FC with context‐footshock pairing in the training trial (upper left) was followed by context re‐exposure and freezing assessment in the FC test (upper right). Visually cued FC was performed in a modified contextual FC chamber wherein the printed gratings that could be rotated were placed over the top of the chamber (bottom). C, Visually cued FC training session is in progress

The VFC system described herein was modified from a commercially available system^4^ (O'Hara & Co., Ltd.) and set in a dim and calm room (80 lux, 50 dB white noise). A sound insulating box (white walls, 60 cm deep × 50 cm wide × 53 cm high) equipped with an overhead charged‐coupled device camera and open‐top transparent cylinder (diameter, 30 cm) with printed black standing bars was placed over the electric‐shock chamber (15 cm deep × 17 cm wide × 13 cm high). The column was guided by a round rail and rotated to make the gratings move at a frequency of 0.5 Hz from right to left (0° angle, square‐wave gratings, spatial frequency of 0.05 cycle/degree, 10°/s). The 18 black bars (2.6 cm each, spaced by the same width of gap) are printed on thin, clear OHP films for laser printing. The film was fixed on cylindrically rolled plastic plates. Cylindrical bars were put in a smooth circular groove, working as the guide rail, of a plastic bucket lid devoid of O‐shaped rubber rings. The rotation timing was controlled automatically by FZ software via a control box (DC‐SB, O'Hara) in coordination with a camera and regulator for electric footshock (SGA‐2010, O'Hara). The control box worked as an electrical outlet and turned an ordinary tube rotator on and off, which horizontally rotated and reeled up a nylon thread rolled around the moving cylinder. The thread, which went through a small hole at the insulator box (with white noise inside), was worked to make a distance from the rotator to the chamber and not to make the sound of machine movement reach the mouse. The training session was 25 minutes long. Mice were first allowed to habituate to the context for 22.5 minutes, and subsequently three 30‐s grating movements were carried out at 22.5‐23.0, 23.5‐24.0, and 24.5‐25 minutes, respectively. Accompanying footshock (0.2 mA) was delivered for 2 seconds, starting at 23 seconds into each 30‐s movement period (Figure 1A).

**FIGURE 2:**
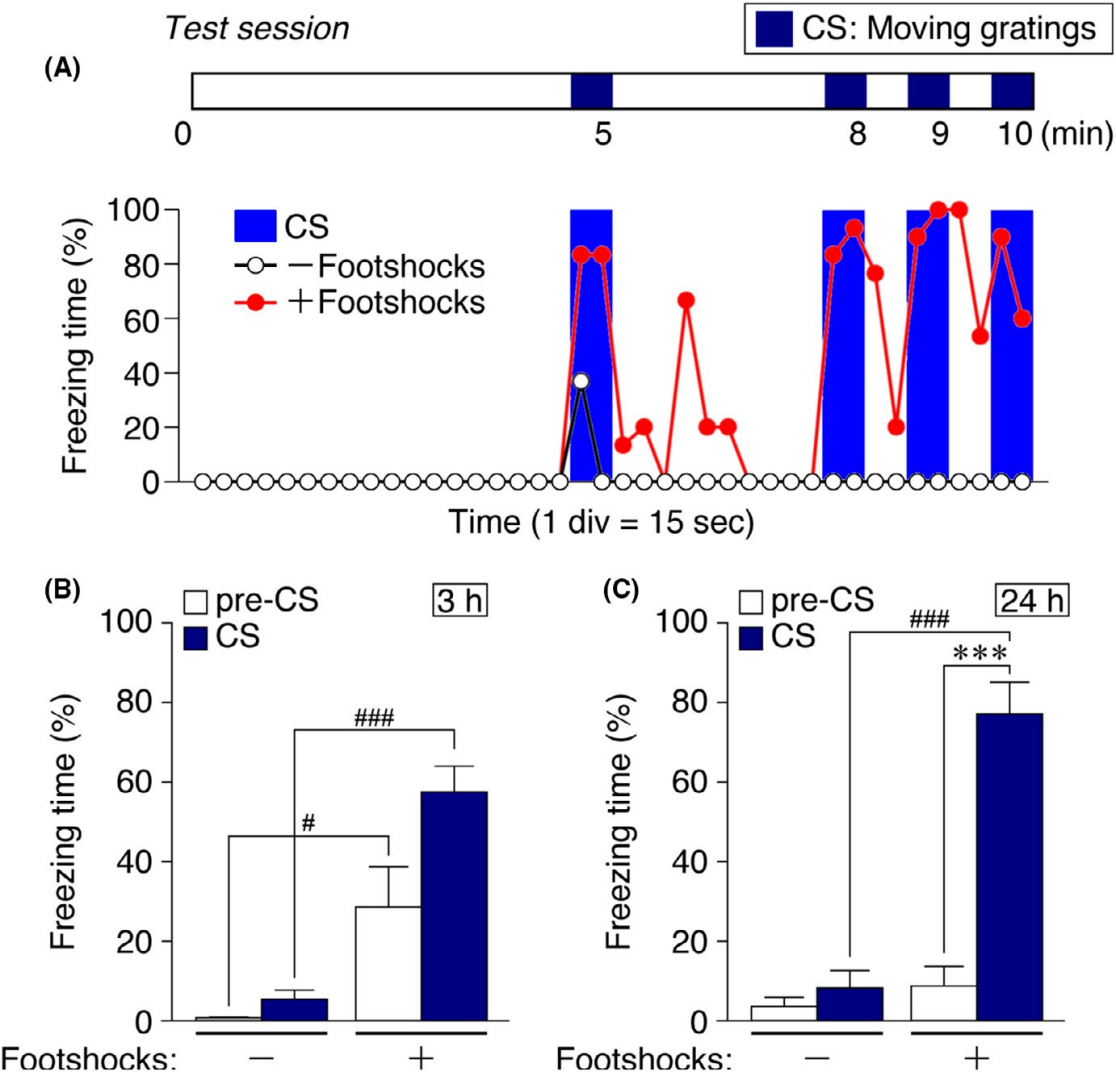
VFC test results. Freezing data (% freezing time per 15‐s interval) from two mice (representative examples) subjected to visually cued FC (red line) in which gratings were moved during 24‐h retention testing with four 30‐s CS delivery periods. Results from sham‐conditioned mice (no US at training) are shown (black line) for comparison. The bars above show the timing of the grating movements (dark blue), which concluded 5, 8, 9, and 10 min into the test session. B and C, Mean freezing scores from sham‐conditioned mice (no shocks in training, left two bars) and conditioned mice (right two bars) for the first 4.5 min of the test session (pre‐CS, white bars) and for four CS delivery sessions (4 × 30 s, dark blue bars) are shown. Significantly increased freezing during CS delivery, relative to the pre‐CS period, was observed when the test was conducted 24 h (C), but not 3 h (B), after the conditioning session. Mean ± SEMs; N = 4 in B, and N = 6 in C; #*P* < .05, ###, ***P < .001, Student's *t* test

For the 10‐min test session, thick white papers replaced metal grids as the floor of the chamber. Four 30‐s grating‐movement periods were administered at 4.5‐5.0, 7.5‐8.0, 8.5‐9.0, and 9.5‐10.0 minutes (Figure 2A). The behavior was recorded with a digital camera and processed for freezing time automatically according to the default setting in FZ software, wherein freezing was recognized as differences in the animal's body image (~5000 pixels), which included <20 pixels over 2 seconds (four serial image frames).

### 2.3 Statistical methods

Microsoft Excel was used for statistical analyses. The differences between groups were compared using Student's t tests. All data are expressed as mean ± SEM. The differences were considered statistically significant when *P* values were <.05.

## 3 RESULTS

### 3.1 VFC

To minimize the effect of contextual FC, mice were habituated to the context by being placed in the context with still gratings for over 20 minutes. Initially, in preliminary experiments, mice were given 10 or 15 minutes to habituate to the apparatus, based on prior descriptions of novel environment exploration proceeding for ~10 minutes^5^. However, some mice required more time to cease exploration and were therefore left to view the training context for a longer period, that is, 22.5 minutes (Figure 1A‐C).

To determine the appropriate delivery parameters for CS–US pairing, variable pairing relationships were explored. To ensure CS–US pairing, we adopted a three‐pairing protocol. By the third pairing, most mice showed freezing, while the rest were often oriented toward the moving gratings, lifted their heads, and occasionally even turned their necks to track the moving speed of the gratings (Figure 1C).

For the retention test, we first explored how mice responded to being replaced in the training box relative to the timing of CS delivery (ie, grating movement). Some mice exhibited freezing only with a delay of several minutes after the first CS delivery. That is, mice appeared to often need some time to recall the CS–US relationship. Thus, after testing various timing protocols, we determined that freezing could be observed regularly if CS was once delivered in the middle of a 10‐minute test trial.

As shown in Figure 2A, when this testing protocol was administered 24 hours after training, greater freezing was observed during CS delivery (30 seconds of grating movement, repeated four times) relative to other time periods, suggesting that the mice formed an association between the distinct visual CS of moving gratings and US, rather than a general association with the context. However, when the test was administered only 3 hours after training (Figure 2B), although conditioned mice froze more than sham‐conditioned mice, freezing during CS delivery was not significantly greater than that observed in the absence of CS within the FC context. Hence, the VFC effect could be captured with 24‐hour long‐term memory testing (Figure 2C, stars). The underlying reason for this time dependence is unknown. It could be that the longer delay allowed the mice to complete sleep‐dependent memory consolidation processes.^6^, ^7^, ^8^

### 3.2 Feasibility of the VFC to genetically modified mice

**FIGURE 3:**
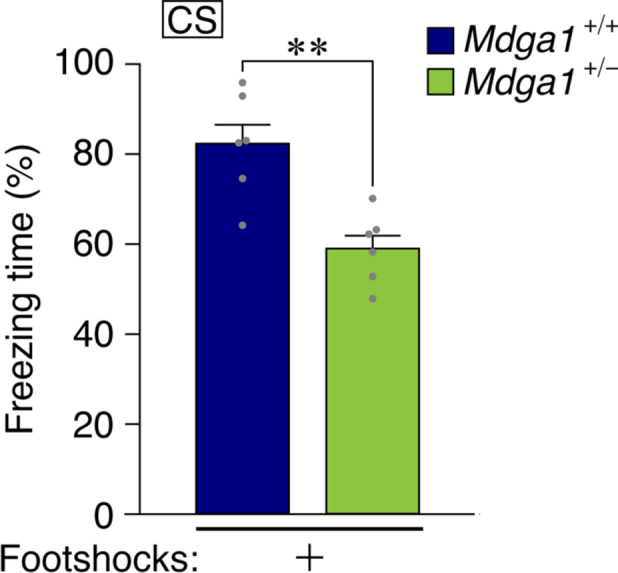
The heterozygous *Mdga1* mutants showing impaired responses in the VFC test. Mice with heterozygous null‐mutant for the *Mdga1* gene (*Mdga1*+/‐) and wild‐type littermates (control; *Mdga1*+/+) were subjected to the VFC test after 24 h of conditioning. Results of freezing scores are expressed as mean ± SEM; N = 6 mice; **P < .01 vs control, Student's *t* test

MDGA1 is a binding protein of neuroligin‐2 that have a role to modulate balance of excitatory‐inhibitory synapses.^9^ The MDGA1 has been reported to have SNPs related to susceptibility to the schizophrenia.^2^ Its null‐mutant mice showed learning deficit in the contextual FC, water maze,^3^ and prepulse inhibition of the startle response.^10^ We tried to apply this new method for learning in the heterozygous *Mdga1*‐mutant mice in which the amount of MDGA1 becomes around half.^11^ The result indicates that these heterogenic mutant mice showed deficit also in this paradigm of learning (Figure 3).

## 4 DISCUSSION AND CONCLUSIONS

Mice can be conditioned to specific visual stimuli in the fear conditioning paradigm. The validity of the test was confirmed in *Mdga1*‐deficient mice. These results may be attributed to the conditions of stimuli because frequencies and other parameters of gratings were set to maximize neuronal responses in mice according to prior research describing visually evoked local field potentials.^12^, ^13^ In humans, visual perception has been studied mainly using fMRI and there is a peak in the responses at lower frequency (coarser) gratings similar to the pattern of mice. Among humans, patients with schizophrenia showed markedly reduced activations to low (coarse), but not high (fine), spatial frequencies relative to control subjects.^14^ Albeit further analyses are warranted, the result obtained here by low‐frequency grating in *Mdga1*‐deficient mice may reflect that the feature in schizophrenic patients that the coarse gratings are less detectable in visual perceptions of the schizophrenic brain.

Here, we conclude that this visually cued fear conditioning paradigm with moving gratings is a useful tool to evaluate animal cortical functions.

## CONFLICT OF INTEREST

The authors declare that they have no conflicts of interest relevant to the content of the article.

## AUTHOR CONTRIBUTIONS

KK, YS, KK, NF, DO, YK, and TY performed experiments. MKY conceived and designed research, performed experiments, analyzed data, and wrote the paper.

## ANIMAL STUDIES

All experimental procedures were carried out in accordance with the Institutional Guidelines and were approved by the Animal Experiment Committee of Tokushima Bunri University.

## Data Availability

The data that support the findings of this study are openly available in Open science framework (OSF) at https://osf.io/7d3gh/ or http://doi.org/10.17605/OSF.IO/U92CP.^15^
